# Scan-Rate-Induced Transition from Redox to Ion Current Rectification in Carbon Nanopipettes

**DOI:** 10.1021/acs.analchem.6c00702

**Published:** 2026-04-24

**Authors:** Gregorio Laucirica, Antonino Biagio Carbonaro, Matteo Nuzzo, Gastón A. Crespo, María Cuartero

**Affiliations:** † UCAM-SENS, Universidad Católica San Antonio de Murcia, UCAM HiTech, Avda. Andres Hernandez Ros 1, 30107 Murcia, Spain; ‡ Department of Chemistry, School of Engineering Science in Chemistry, Biochemistry and Health, KTH Royal Institute of Technology, Teknikringen 30, SE-114 28 Stockholm, Sweden

## Abstract

The electrochemical performance of carbon nanopipettes results from the interplay between ion transport and electron transfer, two distinct charge-based processes governed by different mechanisms that offer complementary insights. We present a simple yet effective approach to decoupling these contributions via cyclic voltammetry. At scan rates below 0.1 V s^–1^, the response is typically driven by redox reactions at the nanopipette surface, as indicated by the presence of redox peaks, whereas at scan rates above 0.5 V s^–1^ up to 1 V s^–1^, the response is dominated by ion transport. Thus, by adjusting the scan rate, it seems possible to transition between electron and iontronic signals. Importantly, this method enables the observation of ICR in the 0.5–1 V s^–1^ range in asymmetric and charged nanofluidic devices, thereby achieving the transition from pure redox behavior to ICR. This tunable strategy opens the door to studying complex systems in nanoelectrochemistry and developing sensors that exploit both signals for different purposes within a single setup.

## Graphical abstract

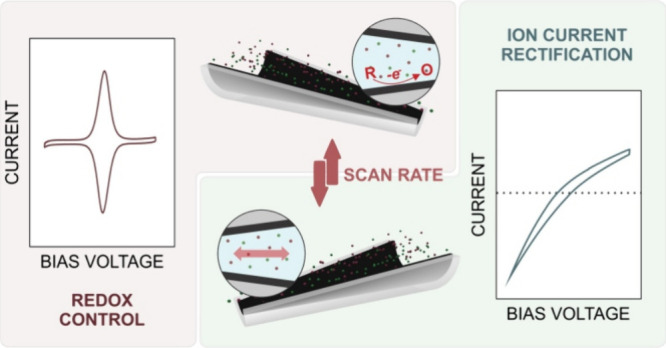

Nanofluidic devices can record two types of signals: iontronic and electronic.^1^ In iontronic measurements, a nanopipette filled with electrolyte and equipped with Ag/AgCl electrodes is used to apply a transmembrane voltage, driving ion transport through the tip.^2^ When the pipet’s dimension falls within the nanometric range (<100 nm), electrostatic interactions become significant, and iontronic measurements may reveal surface properties such as charge, wettability, and effective tip size.^3^ Under certain conditions, these systems can exhibit ion current rectification (ICR), a transport regime that is particularly sensitive to surface charge and widely used for surface characterization and sensing.^4–6^

When using conducting nanopipettes, such as glass pipettes internally modified with a carbon layer (carbon nanopipettes, CNPs), acting as the working electrode of an electrochemical cell, the signal is primarily governed by redox reactions occurring on its surface.^7,8^ Given the narrow dimensions of the CNP, the electrochemical response is featured by a thin-layer regime, which has attracted considerable attention due to its prospects in the development of calibration-free sensors.^7,9^ Accordingly, iontronic and electronic outcomes provide different information, making nanofluidic devices highly versatile for various applications and fundamental studies. While iontronic measurement mainly reveals more direct insight into surface properties, electronic signals offer information about the redox probe (in solution or CNP walls).^9^ In this context, the ability to obtain both types of signals from a single setup is highly advantageous.

In this context, electrochemical impedance spectroscopy (EIS) enables the deconvolution of iontronic and electronic contributions in CNPs, because of the distinct characteristic times of ion transport and redox processes.^10,11^ This makes EIS a promising technique for simultaneously capturing both signals.^12^ However, several challenges are present: (1) instrumental limitations when measuring high-impedance nanofluidic systems (e.g., highly tapered nanopipettes or low-supporting electrolyte conditions); (2) the interpretation of EIS data is often based on equivalent circuit modeling, which relies on a detailed understanding of the underlying physicochemical processes; and (3) the strong dependence of ion transport and redox reactions on the applied bias, necessitating measurements at multiple direct current potentials. This increases experimental time and may compromise system stability.

In this Letter, we propose a rapid and simple alternative for obtaining information about ion and electron transfer in CNPs by employing cyclic voltammetry (CV). In contrast with traditional ion transport measurements, this strategy does not require backfilling the pipet with different solutions, as each process can be probed within the same experiment by scanning the voltage at different frequencies (scan rates), without the need for multiple experimental setups. CNPs were fabricated in a two-step process, where quartz capillaries were pulled to form glass nanopipettes with a nanometric orifice (approximately ∼65 nm radius) and then, a carbon layer was deposited via chemical vapor deposition (details in Section S1 and Figure S1 in the Supporting Information). The conductive properties of the carbon layer allowed the use of the CNP as the working electrode of the electrochemical cell, with an Ag/AgCl wire serving as the pseudoreference auxiliary electrode in a two-electrode configuration. Notably, post-grafting of the carbon surface with *para*-aminobenzoic acid (*p*-ABA) introduces abundant −COOH groups that induce ion current rectification (ICR), which can be readily revealed by tuning the CV scan rate. Overall, these findings open new avenues for both CNP characterization and their application in sensing and nanoelectronics.

## Results and Discussion

Figure 1a shows the CV observed at 0.05 V s^–1^ with the CNP immersed in an aqueous 0.3 M KCl (pH 6) solution containing 1 mM hydroquinone (HQ) (further information on the rationale behind the selection of HQ for the electrochemical experiments and its redox behavior is provided in the Supporting Information (Section S1)). At this sweep rate, the CV response showed the typical peak pair for HQ at ∼0.05 V due to the oxidation of HQ to benzoquinone (BQ). The peak potential separation (Δ*E*_p_) was 25 mV, quite below the typical 59 mV for bulk solution experiments, which is indicative of a thin-layer regime. Ideally, Δ*E*_p_ should tend to 0 mV as the behavior approximates a pure thin-layer effect. The reduction in the Δ*E*_p_ further suggests that, on the time scale of the experiment, the analyte was exhaustively consumed inside the nanofluidic device. Under these conditions, the voltammetric peak charge can be related to the number of moles through Faraday’s law, allowing the estimation of the solution volume inside the CNP, given that the redox probe concentration is known.^13^ In our experiment, the volume was calculated to be 3.6 ± 0.3 pL from the oxidation peak in the first cycle of the CVs at 0.025, 0.05, and 0.075 V s^–1^. Notably, as the scan rate increased beyond 0.05 V s^–1^, Δ*E*_p_ displayed an evident increment. For example, Δ*E*_p_ reached 136 mV at 0.5 V s^–1^ and 546 mV at 5 V s^–1^, corresponding to increases of more than 5-fold and 20-fold, respectively, relative to the value measured at 0.05 V s^–1^. This trend stems from the uncompensated resistance due to the limited ion transport through the narrow tip and is accompanied by deviations from the Gaussian peak shape expected for thin-layer systems. Both peak shift and distortion become more pronounced with increasing scan rate, and at scan rates above 20 V s^–1^, the peaks vanish, yielding a linear, ohmic response.

**Figure 1:**
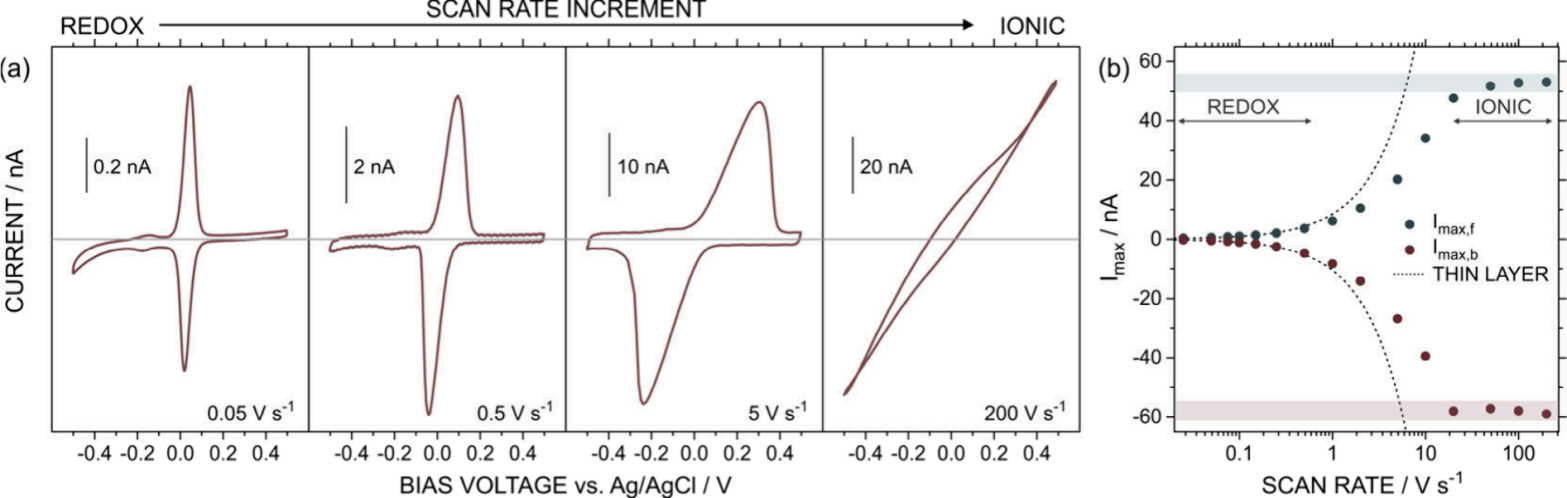
(a) Cyclic voltammograms at different scan rates. The 0 nA current value is indicated with a line. All the measurements were conducted in an aqueous solution of 1 mM HQ in 0.3 M KCl at pH 6. (b) Diagram showing the maximum currents (*I*_max_) in the voltammograms at the different scan rates. *I*_max,f_ and *I*_max,b_ are the maximum currents obtained in the forward (from −0.5 to 0.5 V, oxidations) and backward scan (from 0.5 V to −0.5 V, reductions), respectively. Dotted lines present the expected current value if *I*_P_ maintains the linear trend with the scan rate obtained at low sweep rates, i.e., the expected value in thin-layer regimes.

Figure 1b depicts the maximum current values (*I*_max_) obtained in the voltammograms recorded at the different scan rates. For those voltammograms where the peaks appeared in the scanned voltage window (low scan rates), *I*_max_ coincided with peak current (*I*_P_). In the voltammograms where any peak was discerned (high scan rates), *I*_max_ corresponded to the current values at ± 0.5 V. The *I*_P_ (and, consequently, *I*_max_) value increased with the scan rate; however, it only followed the linear trend predicted in the thin-layer theory below 0.25 V s^–1^ (for clarity, the results provided in Figure 1b are available in Figure S2 on a linear scale). Similar to the trend observed in the peak potentials, this is ascribed to the peak distortion (loss of the Gaussian shape) caused by the CNP’s resistance, which was accentuated as the scan rate was increased. Thus, the scan rate increment led to an increase in the half-height wide peak and a decrease in *I*_P_ (regarding the linear increment expected).^9^

When the scan rate was significantly high (e.g., >10 V s^–1^), the CV did not display any discernible peak, and further increments in the scan rate did not yield any notable change in the response. Under this condition, ion transport became the limiting-rate step, and the response turned fully iontronic. Thus, the CV denoted a nearly linear response proper of an ohmic regime with a voltage-independent resistance of ∼8.5 MΩ. Such curves did not evidence significant changes with further increments in the scan rates, e.g., *I*_max_ changed less than 5% between 50 and 200 V s^–1^ (Figure S2).^14,15^ Furthermore, the position of the curves and the zero-current potential varied, depending on the redox probe in solution (Figure S3). Notably, the ion current values obtained by the CV at high scan rates agree with those obtained by EIS, as demonstrated by the overlap of both plots in Figure S4. Therefore, the signal can be switched from redox-controlled (electronic) to ion-controlled (iontronic) by simply setting the scan rate magnitude.

The combination of surface charges provided by the carbon layer and the geometrical asymmetry of CNP generates a disruption in the electrical potential symmetry that gives rise to ICR.^4,16^ However, this phenomenon is typically observed at moderate to low supporting electrolyte concentrations, where surface charges are not effectively screened by the ions and therefore play a central role in governing ion transport behavior. For this reason, the CNP’s response at low supporting electrolyte concentrations was also evaluated, and the results are presented in Figure 2.

**Figure 2:**
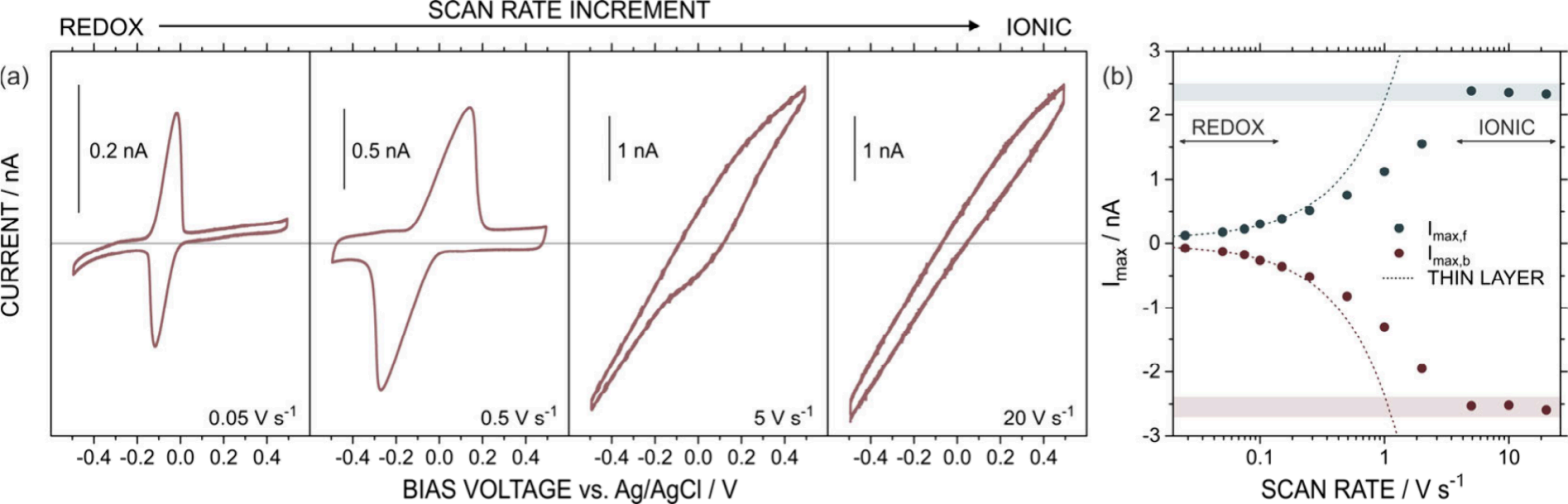
(a) Cyclic voltammograms at different scan rates. The 0 nA current value is indicated with a line. All the measurements were conducted in an aqueous solution of 1 mM HQ in 0.01 M KCl at pH 6. (b) *I*_max_ obtained from the voltammograms at the different scan rates. Dotted lines display the expected current values in an ideal thin-layer regime.

The cyclic voltammograms obtained with low supporting electrolyte concentration, such as 1 mM HQ in 0.010 M KCl at 0.050 V s^–1^, displayed the expected pair of peaks due to the redox reaction of HQ in the thin-layer environment of the CNP (Figure 2a). Applying the Faraday law (see Section S1 in the Supporting Information), the volume in this experiment was estimated to be 1.75 pL. Also, the Δ*E*_p_ value was around 40 mV, which was slightly higher than the value obtained in 0.3 M KCl. This deviation is consistent with expectations, as reduced solution conductivity exacerbates ion transport limitations, thereby increasing peak separation. Moreover, the increment of the scan rate produced a similar effect to that previously explained. For instance, Δ*E*_p_ incremented from 40 mV to 410 mV by increasing the scan rate from 0.050 V s^–1^ to 0.50 V s^–1^. For comparison, the same scan rate range in 0.3 M KCl resulted in a 5-fold increase in Δ*E*_p_, from 25 mV to 136 mV. *I*_P_ also increased with the scan rate following a linear trend for sweep rates <0.1 V s^–1^ (Figure 2b). At higher scan rates, peak distortion attenuated the rate of current increase typically expected for thin-layer systems. Above 5 V s^–1^, the cyclic voltammogram did not exhibit the typical redox peaks and demonstrated a pure ionic response. Under these conditions, the curve displayed a slightly asymmetrical shape with higher conductance values at negative bias voltages. Further increments in the scan rate did not generate appreciable variations in the currents.

Overall, by analogy with EIS, where ion-transport processes occur at different frequencies than electrical double-layer charging and redox reactions, the choice of scan rate allowed the isolation of a purely iontronic signal, a mixed response, or an (almost pure) electronic signal. Since the balance between iontronic and electronic contributions depends on their relative weights, the scan rate at which each process becomes dominant strongly depends on the experimental conditions. In particular, a pure iontronic signal was observed at lower scan rates when ion transport was hindered with more resistive solutions. Thus, reducing the concentration of the supporting electrolyte not only induced a weak ICR behavior but also allowed the pure iontronic signal to be detected at lower scan rates. While at 5 V s^–1^, the electrochemical response in 0.3 M KCl still exhibited the characteristic redox peaks of HQ, only the ionic contribution was observed in 0.01 M KCl, within the same scanned voltage window (see Figures 1a and 2a).

To further illustrate this behavior, Figure 3 shows the response of the same CNP in 1 mM HQ and 0.010 M KCl for different inner volumes. As the volume increased, the amount of HQ involved in the reaction increased, enhancing the faradaic (electronic) contribution at a given scan rate. This was evident from cyclic voltammograms recorded at 0.05 and 0.5 V s^–1^ (Figure 3a), where the current increased with scan rate across all volumes. For the smallest volume, a 10-fold increase in scan rate led to changes in peak separation (from 98 to 410 mV) and *I*_P_ (from 0.17 to 0.76 nA), while maintaining well-defined, bell-shaped peaks characteristic of thin-layer behavior. In contrast, at 7.5 pL, the redox peaks could not be fully captured within the potential window at 0.5 V s^–1^. At the largest volume (28 pL), the response was dominated by ion transport, showing a slightly asymmetric iontronic signal consistent with cation-driven rectification at a negatively charged surface. Overall, these results indicate that lower scan rates are required to isolate the iontronic contribution as the volume increases.

**Figure 3:**
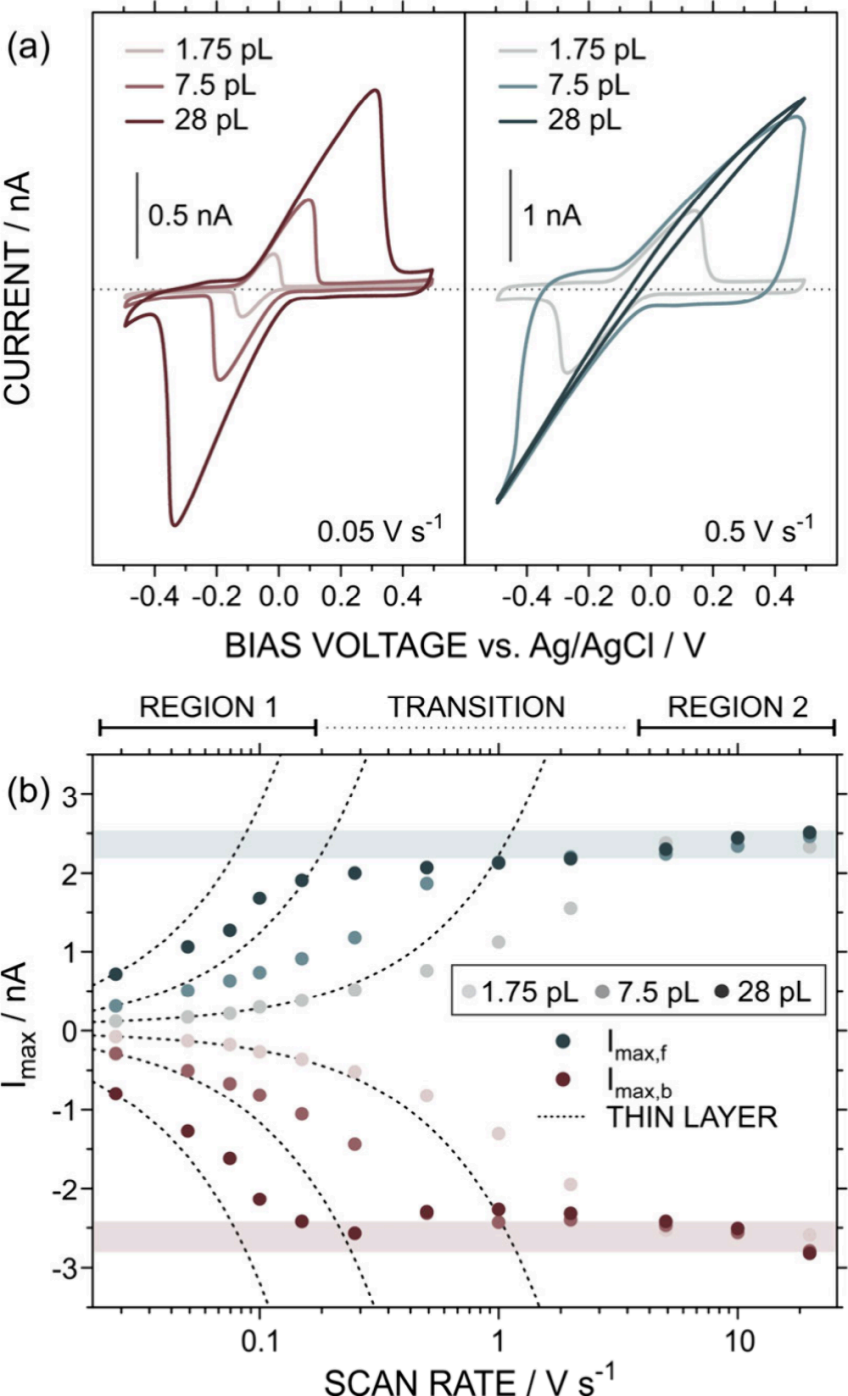
(a) Voltammograms at 0.05 V s^–1^ (left panel) and 0.5 V s^–1^ (right panel) at three different inner volumes. Dashed lines indicate the 0 nA current value. (b) *I*_max_ extracted from the voltammograms at the three different volume conditions vs the scan rate. Dashed lines display the expected values in an ideal thin-layer regime. All the measurements were conducted in an aqueous solution of 1 mM HQ in 0.01 M KCl at pH 6. To guide the reader, “REGION 1”, “REGION 2”, and “TRANSITION” were marked considering the results obtained for 1.75 pL.

The effect of confined volume on the electrochemical response was further analyzed through the dependence of *I*_max_ on the scan rate (Figure 3b), revealing two distinct regimes separated by a transition zone. At low scan rates, the response was dominated by HQ redox behavior, with *I*_max_ corresponding to CV peaks and scaling quasi-linearly with scan rate (REGION 1). At high scan rates (>5 V s^–1^), the redox peaks fell outside the potential window, and *I*_max_ reached a plateau of ∼2.5 nA, corresponding to ion transport-limited current (REGION 2). Between these regimes, a sublinear increase in *I*_max_ was observed due to uncompensated resistance.

The scan rate range of each regime strongly depended on the internal volume. Increasing the volume shifted REGION 1 to lower scan rates (from <0.25 V s^–1^ at 1.75 pL to <0.025 V s^–1^ for >7.5 pL) and promoted the onset of the iontronic regime at lower scan rates (from >5 V s^–1^ at 1.75 pL to 0.5 V s^–1^ for 28 pL). As discussed in previous works, this behavior can be explained, at least in part, by the increase in the number of moles rather than by an increase in CNP’s ion resistance.^9,10^ This fact enhances, in turn, the faradaic signal, which cannot be properly matched by ion migration, because of the high resistance at the tip. As determined by EIS analysis, this phenomenon introduces a time delay in achieving the complete redox conversion of the analyte inside the CNP.^10^

Regardless of the volume, the current measured at scan rates above 5 V s^–1^ was nearly constant (<5% variation), indicating a purely iontronic response governed by volume-independent ion transport resistance, as previously reported.^10^ Once the plateau was reached, further increases in scan rate produced only minor changes (∼10% from 5 to 20 V s^–1^), consistent with previous reports showing that significant variations in iontronic output occur mainly at very high scan rates (>100 V s^–1^).^14,15^

Although large volumes and high redox probe concentrations facilitate the observation of both iontronic and electronic signals, their decoupling can also be achieved in the absence of a redox probe (Figure S5a). In this case, larger electrochemical areas, higher CNP resistance, and much higher scan rates (>10 V s^–1^) are required to ensure that the capacitive electronic current exceeds the ionic current. Additionally, under these conditions, the zero-current potential becomes dependent on the applied voltage window (Figure S5b).

Beyond its applicability, the ability to obtain both signals without modifying either the setup or the solutions is highly advantageous, as it provides a rapid method to characterize surface states. As a proof of concept, Figure 4 shows the response of a CNP recorded at low and high scan rates after electrografting *para*-aminobenzoic acid (*p*-ABA). For the electrografting modification, the CNP was immersed in a previously prepared 18 mM solution of 4-carboxybenzenediazonium chloride (+N_2_–Ar–COOH), generated from the diazotization of *p*-ABA as the aromatic amine precursor. The resulting diazonium salt reacts with the CNP carbon layer upon applying potentials below −0.2 V (Figure 4a and 4b) (Raman and electrochemical characterizations are available in Table S1 and Figure S6).

**Figure 4:**
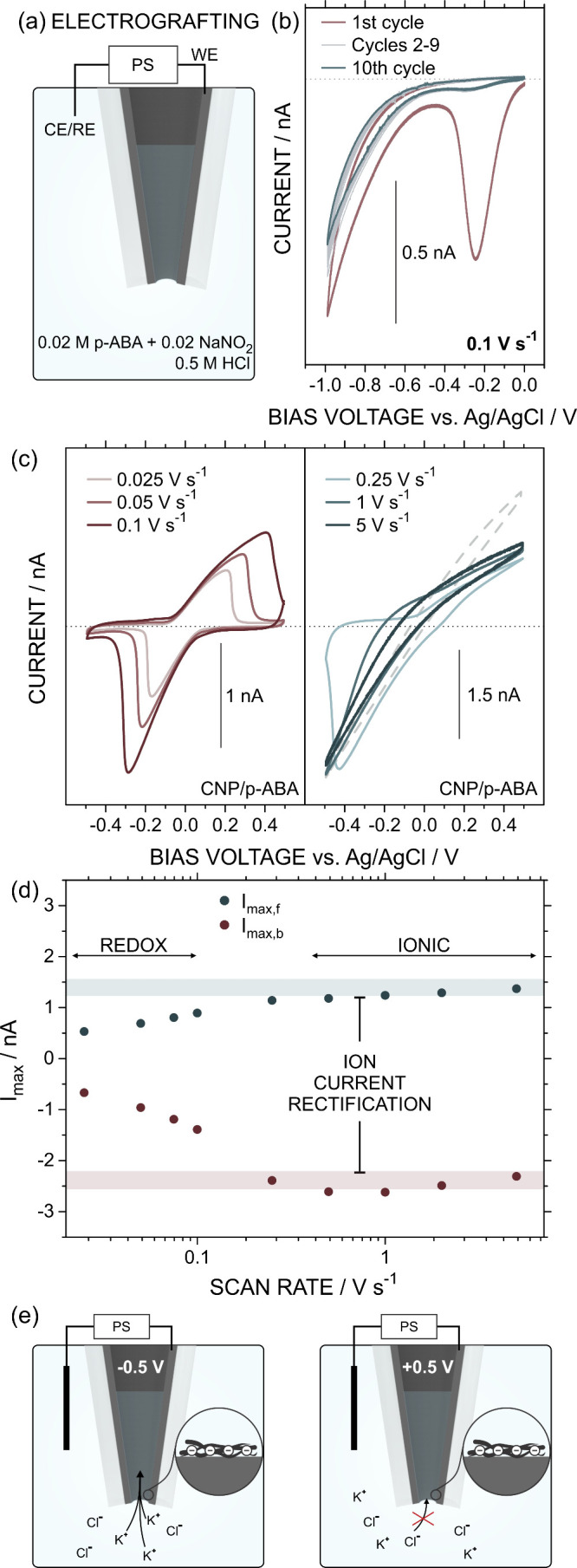
(a) Scheme of the experimental setup and (b) electrochemical response obtained in the electrografting of *p*-ABA. (c) Voltammograms at different scan rates for the *p*-ABA-modified CNP in an aqueous solution of 0.01 M KCl at pH 6. Dotted lines indicate the 0 nA current value. Dashed line in the right panel depicts the CV obtained at 5 V s^–1^ for the CNP before *p*-ABA modification. (d) *I*_max_ extracted from the voltammograms in terms of the scan rate. (e) Scheme illustrating the origin of the ICR phenomenon due to the ion accumulation/depletion obtained at −0.5 V and +0.5 V, respectively. The terms “WE”, “CE/RE”, and “PS” refer to the working electrode, pseudoreference electrode, and power supply, respectively.

At low scan rates (<0.1 V s^–1^), the CV of the *p*-ABA-modified CNP in 1 mM HQ and 0.01 M KCl displayed redox peaks characteristic of the HQ/BQ redox transition (Figure 4c). Ion transport across the CNP tip introduced a significant uncompensated resistance, leading to a peak potential separation of 0.38 at 0.025 V s^–1^, which nearly doubled to 0.609 V upon increasing the scan rate to 0.050 V s^–1^. At scan rates above 5 V s^–1^, the response became purely iontronic. Notably, the high density of −COO– groups at pH 6, combined with the device asymmetry, resulted in a marked increase in ICR efficiency, from 1.08 to 1.80 (see the definition for “rectification” in the Supporting Information) (Figure 4d). Although the enhancement of rectification upon introducing charged groups has been extensively reported and attributed to increased ion accumulation/depletion at opposite polarities (Figure 4e),^17^ this represents, to our knowledge, the first demonstration of a single setup enabling a transition between ICR (pure iontronic signal) and conventional electrochemical CV responses (electronic signal) solely by tuning the sweep rate.

Overall, our findings highlight CV as a versatile tool for distinguishing between electronic and iontronic signals in nanofluidic devices by employing the same experimental setup. The ability to modulate the scan rate to capture redox activity or ion transport behaviors selectively offers significant advantages for electrochemical characterization, particularly in cases where traditional spectroscopic methods are challenging. The transition from redox-controlled responses to ICR also provides valuable insights into surface charge properties and ion transport mechanisms. The ICR regime can be evidenced in the same experiment, without the need to remove the pipet or refill it with a different electrolyte solution, thus providing a straightforward approach with respect to the experimental setup commonly used in ion transport recordings. Therefore, this simplifies the study of nanofluidic systems and expands potential applications in sensing, surface characterization, fundamental studies, and electronic devices. Also, this Letter introduces the study of the CNP modification with *p*-ABA by evaluating the changes that this generates on the surface charged state and, consequently, in ICR. Importantly, considering that the modification of CNPs is not trivial, this strategy provides a clear-cut method for introducing abundant −COOH groups onto the CNP surface. These groups can serve as suitable anchoring sites for the attachment of molecules of different nature, e.g., through carbodiimide coupling chemistry, thereby paving the way for generating different functionalities to the nanofluidic device.

## Supplementary Material



## Notes

Materials and methods; comparison CV vs EIS; spectroscopic and electrochemical characterization of p-ABA film (PDF)
