# Supplementary material for: Scan-Rate-Induced Transition from Redox to Ion Current Rectification in Carbon Nanopipettes
Source: Anal Chem. 2026 Apr 24;98(18):13197–202. doi: 10.1021/acs.analchem.6c00702 (PMC13177282; doi:10.1021/acs.analchem.6c00702)
Supplement: Supplementary file 1 [file ac6c00702_si_001.pdf]

Supporting information for:

Scan-Rate-Induced Transition from Redox to Ion Current Rectification in Carbon Nanopipettes

Gregorio Laucirica[a], Antonino Biagio Carbonaro[a], Matteo Nuzzo[a], Gastón A. Crespo[a,b], María Cuartero[a,b,*]

[a]UCAM-SENS, Universidad Católica San Antonio de Murcia, UCAM HiTech, Avda. Andrés Hernández Ros 1, 30107, Murcia, Spain.

[b]Department of Chemistry, School of Engineering Science in Chemistry, Biochemistry and Health, KTH Royal Institute of Technology, Teknikringen 30, SE-114 28 Stockholm, Sweden.

*mariacb@kth.se

CONTENT

S1. EXPERIMENTAL DETAILS 3

Materials. 3

Glass nanopipettes. 3

Carbon-coated glass nanopipettes (CNPs). 3

Electrochemical setup. 3

p-ABA electrografting. 6

S2. FIGURES 10

S3. REFERENCES 14

S1. EXPERIMENTAL DETAILS

Materials. Potassium chloride KCl (99.5%), hydrochloric acid HCl (1 N), and sodium nitrite $NaNO_2$ (≥99.0%) were purchased from VWR chemicals. MES hydrate (>99%) was purchased from Thermo Scientific. Hydroquinone HQ (≥99.0%), sodium hypochlorite solution (6–14% chlorine), 4-aminobenzoic acid p-ABA (≥99.0%), and Ag wires (Ø 0.5 mm) were provided by Merck. All the reagents were employed without any further treatment and prepared in ultrapure Milli-Q® water (18.2 MΩ cm at 25°C, Merck Millipore).

Glass nanopipettes. Quartz capillary tubes without filament with an inner diameter of 0.7 mm, outer diameter of 1 mm, and length of 10 cm were obtained from Sutter Instrument (Novato, CA). The nanopipettes were fabricated from these capillaries by employing a CO_2 laser-based puller P-2000 by Sutter Instrument. The program was the following:

Heat	Filament	Velocity	Delay	Pull
700	4	60	145	175

Scanning electron microscopy (SEM, Apreo S, Thermo Fisher Scientific) analysis suggested tip radii around 65 nm (**Figure S1**).

Carbon-coated glass nanopipettes (CNPs). The carbon layer was deposited by chemical vapor deposition (CVD).[1] The method consisted of exposing the glass nanopipettes to a gas mixture of CH_4:Ar 0.2:0.6 L min^{-1} at 925 °C for 3.5 minutes. The carbon layer is deposited only on the inner surface of the CNP, as demonstrated by the thin-layer CV.

Electrochemical setup. All electrochemical experiments were done by using a two-electrode setup consisting of a homemade Ag/AgCl wire as the auxiliary electrode (CE/RE) and the CNP as the working electrode (WE). The Ag/AgCl wire electrode was prepared by immersing a 5 cm length Ag wire into a diluted sodium hypochlorite solution

for 1 hour. The necessary electrical connection between the nanotip (WE) and the potentiostat socket was made by inserting an Ag wire on the back side of the pipette. The electrodes were positioned at approximately 1 cm inside the measurement reservoir.

For the CV experiments, the electrodes were connected to a HEKA amplifier operated with the PatchMaster software®. For the EIS experiments, the electrochemical cell was connected to a VIONIC potentiostat from Metrohm® operated with the Intello 1.5 software. All the experiments were conducted inside a Faraday cage (Rittal, GmbH & Co. KG).

CV experiments were performed at different scan rates. The potential window was typically set to ±0.5 V, in accordance with voltage ranges commonly used in iontronic experiments. Potentials beyond this window were avoided because, at low scan rates under electrochemical control, side reactions could generate large currents, such as water electrolysis at potentials below −0.5 V.

EIS experiments were performed by applying a sinusoidal perturbation with frequencies from 0.1 Hz to $1x10^6$ Hz (ten points per decade) and an amplitude of 10 mV. The sinusoidal perturbation was superimposed on different direct current potentials (E_{DC}) from -0.2 V to 0.4 V.

Voltammetric charge was determined by the ratio of the peak area in the voltammogram and the scan rate. The voltammetric charge (Q) was employed to determine the volume inside the CNP (V) by applying the following equation (Faraday's Law):

$$Q = nFCV \qquad \text{Eq. S1}$$

Where n, F, and C are the number of electrons in the redox reaction, the Faraday constant, and the redox probe concentration, respectively.

Control of the volume inside the CNP was performed by employing a pressure controller uMc-PPC by SENSAPEX®. Typically, pulses of -70 kPa for 5 seconds were applied to

generate increments in the volume while the CV was running. After the pulse, the current rapidly stabilized and kept a constant cycle-to-cycle.

Hydroquinone (HQ) (pKa~9.9) was selected as the redox probe for these experiments. This choice was primarily motivated by the neutral state of HQ at pH 6, which prevents its accumulation or exclusion from the CNP due to electrostatic interactions with surface charges, especially at low supporting electrolyte concentrations (typically <0.01 M).[2] However, as shown in **Figures S4** and **S5**, the inclusion of a redox probe in the electrolyte solution is not strictly necessary to evidence the iontronic regime. In this work, HQ was added to enable measurements at lower scan rates, where the iontronic contribution can be clearly identified due to the presence of faradaic currents, and to unambiguously demonstrate the transition from the electronic to the iontronic regime. Finally, considering the high CNP resistance at 0.01 M, most of the effects on the CV were explained in terms of this contribution, but it is worth mentioning that the charge transfer resistance of HQ also partially contributed to the peak separation as the scan rate increased.

Notably, the electrochemical response of the HQ/BQ system is known to be sensitive to several experimental parameters, including pH, solvent, buffer composition, supporting electrolyte, and probe concentration, and may involve side chemical reactions under certain conditions.[3–5] For instance, in our experiments, at scan rates ≥ 0.1 V s^{-1}, the oxidation and reduction voltammetric charges differed by less than 5%. In contrast, at lower scan rates, significant oxidation/reduction charge differences were observed: at 0.05 V s^{-1}, the reduction charge was 20% lower than the oxidation charge, which is consistent with the occurrence of side chemical reactions following HQ oxidation. In addition, a small pair of peaks appeared at more negative potentials. This redox transition is usually linked to the variation of the local pH due to the redox switching.[4] These effects, widely discussed for HQ/BQ in the literature, may become further accentuated under the strong confinement conditions of CNPs. To prevent significant pH

changes resulting from the HQ redox reaction, all solutions were buffered with 5 mM MES.

Rectification factor. Rectification factor. As discussed in the main manuscript, the zero-current potential (the so-called reversal potential) may depend on several experimental variables, such as the redox probe or the scanned potential window. In this context, rectification is understood as an asymmetric conductance response with respect to the reversal potential. To quantify rectification efficiency, the rectification factor was introduced and defined as the ratio between the currents measured at the two extremes of the applied voltage window. Notably, shifts in the iontronic curves arising from the nature of the redox probe or, in experiments performed in the absence of redox species, from the selected potential window may affect the magnitude of the rectification factor. Therefore, meaningful comparisons based on this parameter require these variables to be kept constant. Furthermore, in view of potential applications to quantitative analysis, alternative approaches could also be considered, such as using resistance values extracted from the extreme regions of the curves or defining a given redox probe as a reference, in a manner analogous to that utilized in experiments at the interface between two immiscible electrolyte solutions.[6,7]

p-ABA electrografting. For the modification, the CNP was immersed in a freshly prepared mixture of 18 mM p-ABA and 18 mM $NaNO_2$, kept in an ice bath. Briefly, 2 mL of a 36 mM $NaNO_2$ aqueous solution was added dropwise to 2 mL of a 36 mM p-ABA solution prepared in 0.5 M HCl under magnetic stirring. Prior to mixing, both precursor solutions were stored for 1 hour at 4 °C. Throughout the process, all solutions were maintained in ice baths to preserve reactivity. The final mixture was aspirated into the CNP by applying a pressure of –70 kPa for 40 seconds. Subsequently, a CV routine of 10 cycles between 0 and -1 V at 0.1 V s^{-1} was performed using the setup described

above. The solution was then expelled by applying –25 kPa for 20 seconds. This entire protocol was repeated three times.

Beyond the ICR measurements, the modified CNP was characterized by performing Raman measurements (**Figure S6a** and **S6b**) and electrochemical routines (**Figure S6c-f**) in the presence of a charged redox probe. A quartz capillary was completely modified with a carbon layer (inside and outside) by changing the flux ratio of CH_4:Ar from 0.2:0.5 L min^{-1} (the typical ratio employed in the electrochemical experiments) to 0.5:0.2 L min^{-1} at 925 °C for 3 minutes. The resultant carbon-coated capillary was employed as an electrode to perform the electrografting under the same conditions as those explained for the CNP. The Raman spectra between 600 and 2300 cm^{-1} were acquired before and after the chemical modification with a PR-1W from JASCO®. The measurement conditions consisted of a 785 nm laser with a power of 25 mW, 10 seconds of integration, and 20 accumulations.

Both Raman spectra showed two active bands in the first-order spectrum, which are the G band due to the in-phase vibration of the graphitic lattice (the E_{2g} mode of the planar carbon domain) located around 1590 cm^{-1}, and the D band, located around 1320 cm^{-1} due to the symmetric A_{1g} mode[8,9] (**Figure S6a** and **S6b**). In both cases, the first order Raman spectrum was fitted in the region 600 cm^{-1} – 2300 cm^{-1}, by the sum of three functions related to the D (~1320 cm^{-1}), D'' (~1500cm^{-1}) and G (~1590 cm^{-1}) bands; it was found that D'' band fits better with Gaussian function, while D and G fit better with Voight function. The I_D/I_G ratios were calculated with the relative intensity of the D and G bands after the fitting, to exclude the contribution due to the D'' band.[10,11]

The Raman spectrum of the bare CNPs in **Figure S6a** showed a prominent D and G band typical of carbon-based materials, with the peak centre located at 1321 cm^{-1} and 1595 cm^{-1}, respectively. The Gaussian contribution attributed to the D'' band is located at 1489 cm^{-1}. After fitting, the I_D/I_G ratio of 1.33 and the D_{FWHM} of 116 cm^{-1} is indicative of a significant structural disorder of the carbon film deposited on the CNPs quartz surface

by CVD decomposition of the methane at 925°C, suggesting an amorphous-like carbon structure, in which planar carbon domains (graphite-like) are interspersed within a main tetrahedral carbon matrix (diamond-like).[9,12] Following the grafting reaction, although the peak center of both D and G bands did not show significant changes in the grafted CNPs Raman spectrum (**Figure S6b**), considerable modification has been observed in the other diagnostic parameters. In particular, the I_D/I_G ratio increases from 1.33 to 1.49, and the D_{FWHM} also increases from 116 cm^{-1} to 145 cm^{-1}. This behaviour is typically observed following the grafting reaction on graphite/graphene-like materials, suggesting an increase in the structural disorder due to the covalent attachment in the planar carbon domain, by the covalent electron transfer from the $^{\bullet}$Ar-COOH aryl radical, which locally converts the planar carbon to tetrahedral carbon.[13,14] All the diagnostic parameters from the first-order Raman spectrum are summarized in **Table S1.** Although this dataset confirms the covalent attachment of Ar-COOH moieties onto the carbon surface, no further conclusions can be drawn regarding the possible formation of dendrimer-like polymeric structures through successive radical attacks at the ortho position, due to the inherent limitations of the experimental technique.[15]

To complement the information obtained by ICR and Raman measurements, CVs and EIS were performed in the presence of 0.77 mM of the charged redox probe $Fe(CN)_6^{4-}$ in 0.3 M KCl. The CV recorded prior to p-ABA integration displayed the characteristic pair of redox peaks for ferrocyanide at approximately 0.2 V, with a peak-to-peak separation of less than 10 mV, consistent with thin-layer conditions (**Figure S6c**). Following p-ABA electrografting, the CV of the modified CNP showed an increase in capacitive current and a suppression of the ferrocyanide redox peaks. This behavior is attributed to the hindered access of ferrocyanide to the carbon surface, likely due to electrostatic repulsion resulting from the increased negative surface charge introduced by the p-ABA layer. Similar trends have been previously reported in the literature.[2]

EIS measurements at 0 V (outside the faradaic window), where the contribution of the redox probe is negligible, showed no significant change in the semicircle diameter (**Figure S6d**). However, the low-frequency increment in $-Z_{IM}$ due to the double-layer capacitance demonstrated a clear attenuation in the slope after the modification. This fact suggests a loss of the surface capacitance ideality due to the integration of the p-ABA film. EIS measurements within the faradaic window (0.2 V) did not reveal appreciable changes in semicircle diameter prior to CNP modification when compared to measurements at 0 V (**Figures S6e–f**). This outcome is consistent with the behavior of redox probes exhibiting very low charge transfer resistance (i.e., fast kinetics). However, following electrografting, EIS measurements revealed the emergence of a second semicircle at low frequencies, with a diameter exceeding 100 MΩ, indicating the appearance of an additional resistance in the system. This low-frequency semicircle can be attributed to a substantial increase in the charge transfer resistance of ferrocyanide after modification. Together with the CV results, the Nyquist plot reinforces the hypothesis that the p-ABA layer significantly hinders the redox transfer of ferrocyanide, likely due to electrostatic repulsion between the negatively charged moieties.

Overall, the CV results obtained during and after electrografting, the iontronic response, and the EIS data, further supported by Raman spectroscopic analysis, are all in good agreement and consistently support the successful surface modification of the CNP after p-ABA electrografting.

Table S1. First-order Raman spectrum deconvolution data related to bare and grafted CNPs.

Parameter	Bare CNPs	Grafted CNPs
I_D/I_G	1.33	1.49
$D_{position}$ (cm^{-1})	1321	1319
$D''_{position}$ (cm^{-1})	1489	1501
$G_{position}$ (cm^{-1})	1595	1596
D_{FWHM} (cm^{-1})	116	145

S2. FIGURES

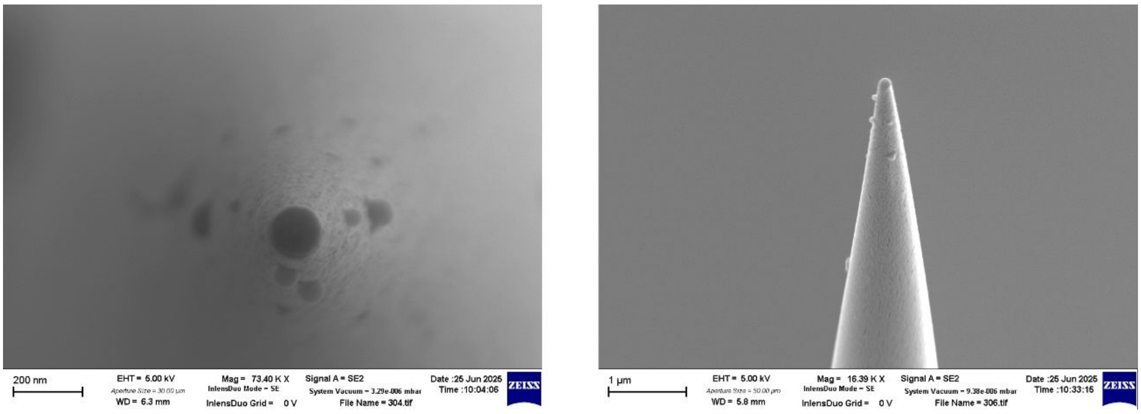

Figure S1. SEM analysis of a CNP obtained at 90° and 0°.

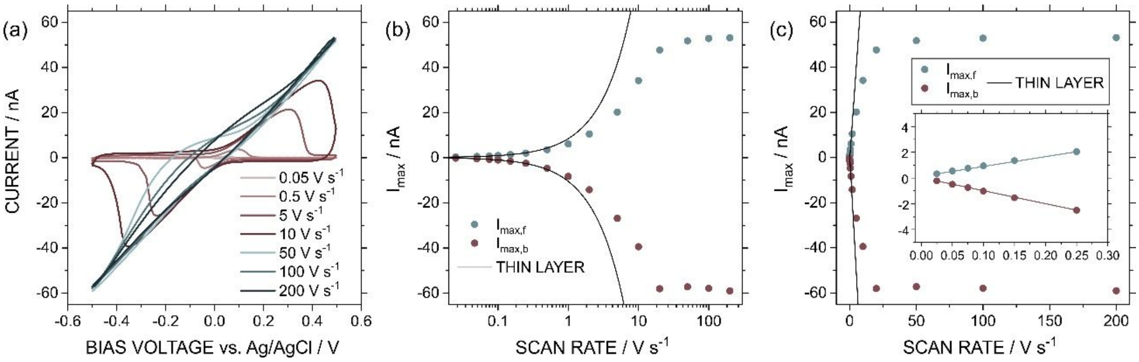

Figure S2. (a) Cyclic voltammograms at different scan rates. All the measurements were conducted in an aqueous solution of 1 mM HQ in 0.3 M KCl at pH 6. Diagrams in (b) logarithmic and (c) linear scales showing the maximum currents (I_{max}) in the voltammograms at the different scan rates. $I_{max,f}$ and $I_{max,b}$ are the maximum currents obtained in the forward (from –0.5 V to 0.5 V, oxidations) and backward scan (from 0.5 V to –0.5 V, reductions), respectively. Lines display the expected current value if peak current (I_P) maintains the linear trend with the scan rate obtained at low sweep rates, i.e., the expected value in thin-layer regimes. Inset in figure (c) demonstrates the linear relationship between I_P and the scan rate at low scan sweeps.

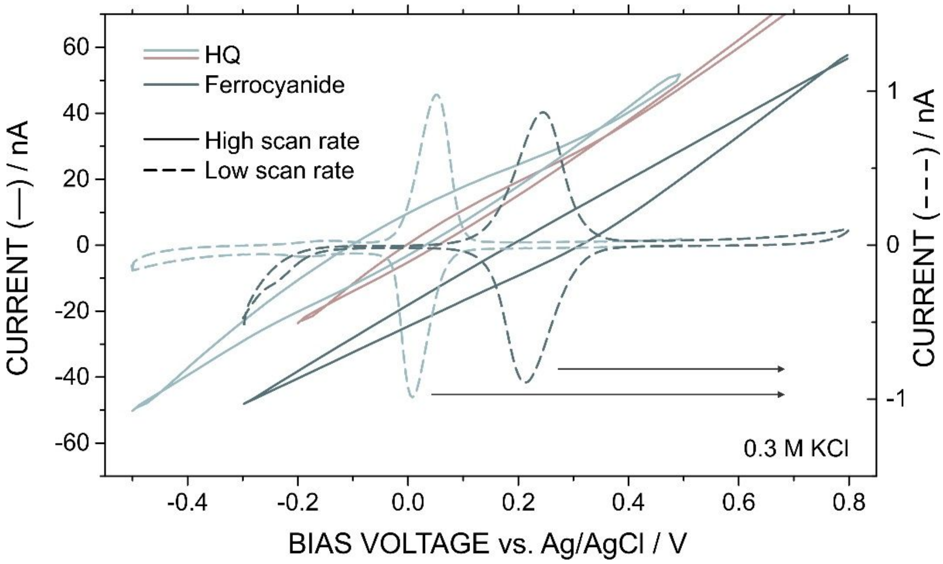

Figure S3. Cyclic voltammograms recorded for two redox probes under low- and high-scan-rate conditions. Measurements were performed in 0.3 M KCl containing either 1 mM HQ or 1 mM $Fe(CN)_6^{4-}$. For HQ, the low and high scan rates were 0.1 and 200 V s^{-1}, respectively. The violet curve corresponds to a repeated measurement at high scan rate but using a different potential window (from −0.2 to 0.8 V). For $Fe(CN)_6^{4-}$, the low and high scan rates were 0.025 and 10 V s^{-1}, respectively. In this latter case, the iontronic regime was reached at lower scan rates because a larger solution volume was used in the experiment.

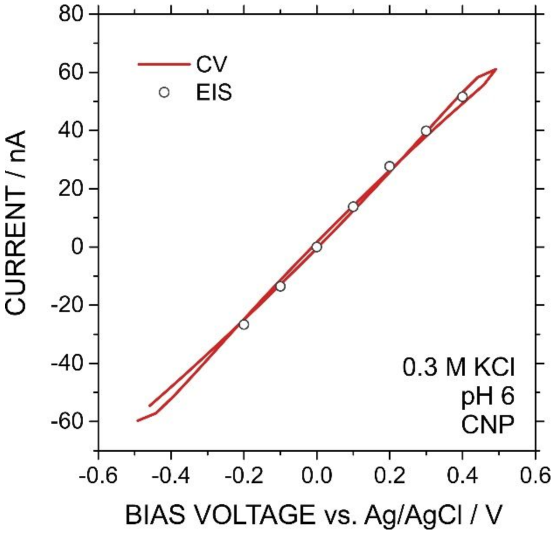

Figure S4. The red lines show CVs of the CNP, while void circles indicate the estimated current from EIS at the different voltages. To ensure a full ion-governed response, the CV was performed at 500 V s^{-1}. The figure compares the CVs obtained at 500 V s^{-1} in the absence of any redox probe (under pure iontronic contribution) and the current values predicted by EIS for an unmodified CNP. The ion resistance (R_{CNP}) was determined at different E_{DC} from the EIS at 0.3 M KCl by estimating the semicircle diameter in the Nyquist plot.

Considering the applied E_{DC} voltage and the obtained resistance value, the theoretical current (I) was determined by Ohm's law:

$$E_{DC} = R_{CNP} I \tag{Eq. S2}$$

As demonstrated by the overlapping of the CV and EIS points, there is a good agreement between the values obtained for both techniques. However, for the case of the CV, the curve was obtained in less than 5 seconds, while in the EIS, it involved obtaining one spectrum (approximately □1.5 minutes of duration) at each E_{DC}.

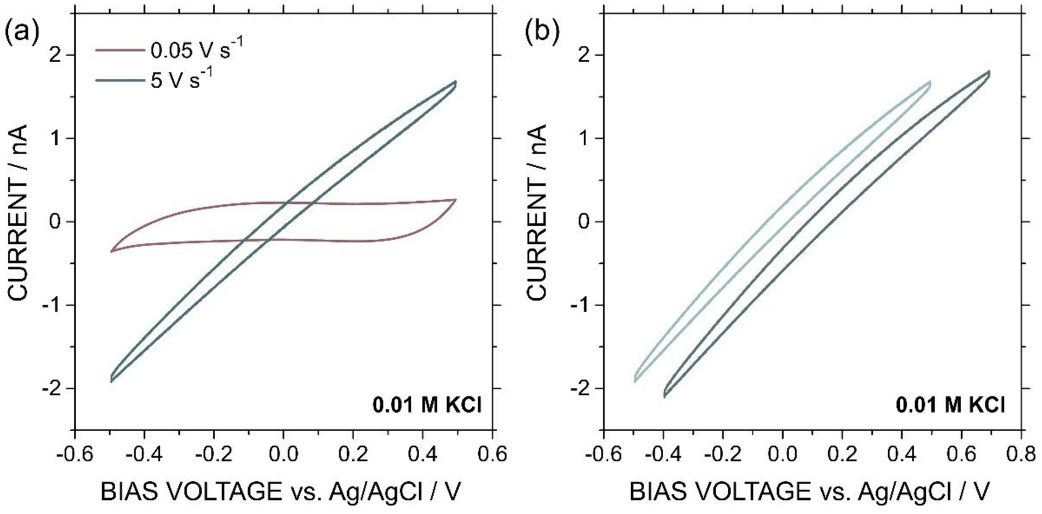

Figure S5. (a) CVs at two different scan rates. (b) CVs recorded at 5 V s^{-1} in two different voltage windows. All the measurements were performed in a bare CNP exposed to 0.01 M KCl.

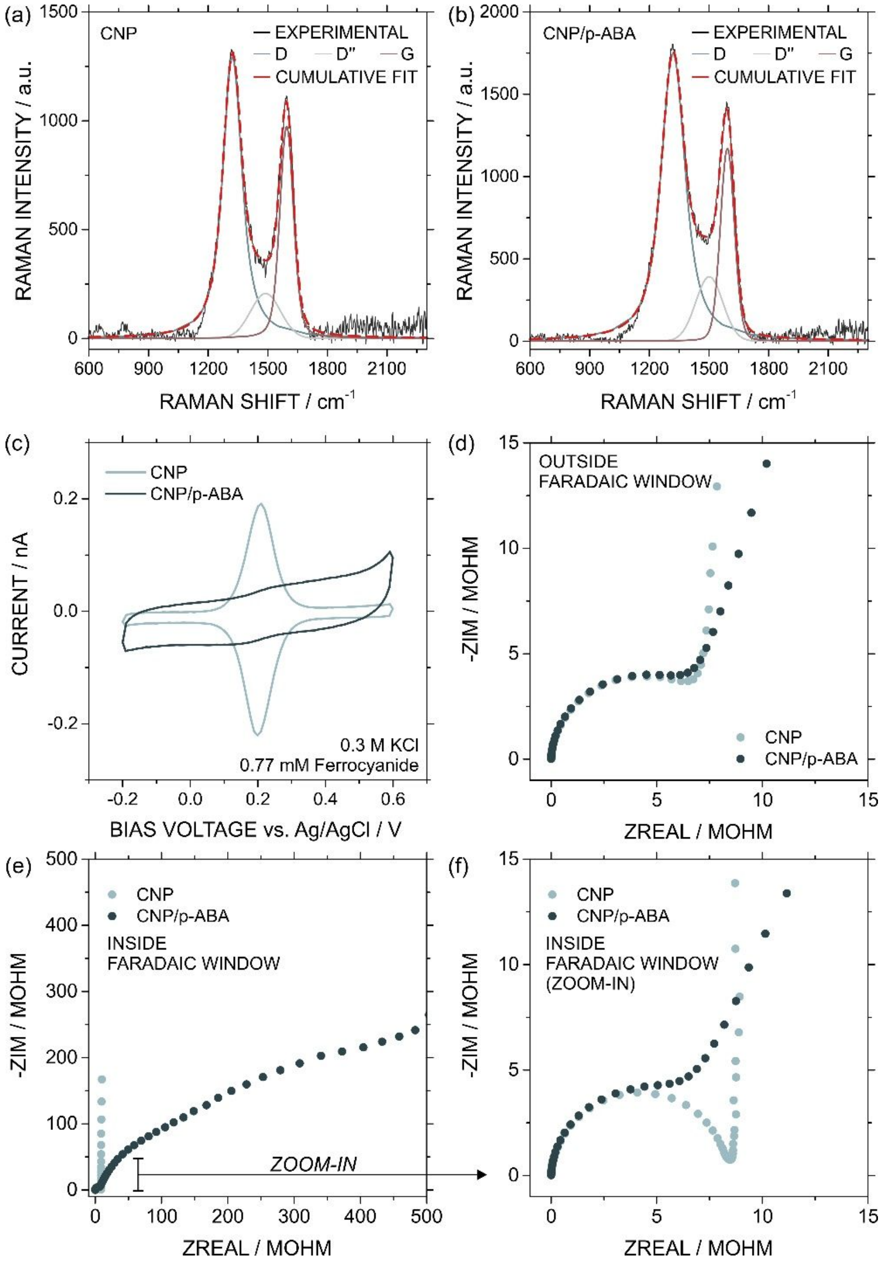

Figure S6. Characterization of p-ABA-modified CNP. Experimental Raman spectra (black line) of the (a) bare CNP and (b) p-ABA-modified CNP. The graphs also show the three-component peak deconvolution. (c) CV of the CNP before and after the modification. EIS at (d) E_{DC}= 0 V and (e) E_{DC}= 0.2 V. (f) Zoom-in of the EIS at E_{DC}= 0.2 V. Measurements in plots (c)-(f) were obtained in an aqueous solution of 0.77 mM $K_4Fe(CN)_6$ in 0.3 M KCl, pH=6.

S3. REFERENCES

(1) Singhal, R.; Bhattacharyya, S.; Orynbayeva, Z.; Vitol, E.; Friedman, G.; Gogotsi, Y. Small Diameter Carbon Nanopipettes. *Nanotechnology* **2010**, *21* (1), 015304. https://doi.org/10.1088/0957-4484/21/1/015304.

(2) Bae, J. H.; Wang, D.; Hu, K.; Mirkin, M. V. Surface-Charge Effects on Voltammetry in Carbon Nanocavities. *Anal. Chem.* **2019**, *91* (9), 5530–5536. https://doi.org/10.1021/acs.analchem.9b00426.

(3) Rafiee, M.; Nematollahi, D. Voltammetry of Electroinactive Species Using Quinone/Hydroquinone Redox: A Known Redox System Viewed in a New Perspective. *Electroanalysis* **2007**, *19* (13), 1382–1386. https://doi.org/10.1002/elan.200703864.

(4) Quan, M.; Sanchez, D.; Wasylkiw, M. F.; Smith, D. K. Voltammetry of Quinones in Unbuffered Aqueous Solution: Reassessing the Roles of Proton Transfer and Hydrogen Bonding in the Aqueous Electrochemistry of Quinones. *J. Am. Chem. Soc.* **2007**, *129* (42), 12847–12856. https://doi.org/10.1021/ja0743083.

(5) Uchimiya, M.; Stone, A. T. Reversible Redox Chemistry of Quinones: Impact on Biogeochemical Cycles. *Chemosphere* **2009**, *77* (4), 451–458. https://doi.org/10.1016/j.chemosphere.2009.07.025.

(6) Langmaier, J.; Trojánek, A.; Samec, Z. Use of the 1,1′-Dimethylferrocene Oxidation Process for the Calibration of the Reference Electrode Potential in Organic Solvents Immiscible with Water. *J. Electroanal. Chem.* **2008**, *616* (1–2), 57–63. https://doi.org/10.1016/j.jelechem.2008.01.001.

(7) Colombo, M. L.; McNeil, S.; Iwai, N.; Chang, A.; Shen, M. Electrochemical Detection of Dopamine via Assisted Ion Transfer at Nanopipet Electrode Using Cyclic Voltammetry. *J. Electrochem. Soc.* **2016**, *163* (4), H3072–H3076. https://doi.org/10.1149/2.0091604jes.

(8) Tuinstra, F.; Koenig, J. L. Raman Spectrum of Graphite. *J. Chem. Phys.* **1970**, *53* (3), 1126–1130. https://doi.org/10.1063/1.1674108.

(9) Ferrari, A. C.; Robertson, J. Interpretation of Raman Spectra of Disordered and Amorphous Carbon. *Phys. Rev. B* **2000**, *61* (20), 14095–14107. https://doi.org/10.1103/PhysRevB.61.14095.

(10) López-Díaz, D.; López Holgado, M.; García-Fierro, J. L.; Velázquez, M. M. Evolution of the Raman Spectrum with the Chemical Composition of Graphene Oxide. *J. Phys. Chem. C* **2017**, *121* (37), 20489–20497. https://doi.org/10.1021/acs.jpcc.7b06236.

(11) Brubaker, Z. E.; Langford, J. J.; Kapsimalis, R. J.; Niedziela, J. L. Quantitative Analysis of Raman Spectral Parameters for Carbon Fibers: Practical Considerations and Connection to Mechanical Properties. *J. Mater. Sci.* **2021**, *56* (27), 15087–15121. https://doi.org/10.1007/s10853-021-06225-1.

(12) Schuepfer, D. B.; Badaczewski, F.; Guerra-Castro, J. M.; Hofmann, D. M.; Heiliger, C.; Smarsly, B.; Klar, P. J. Assessing the Structural Properties of Graphitic and Non-Graphitic Carbons by Raman Spectroscopy. *Carbon N. Y.* **2020**, *161*, 359–372. https://doi.org/10.1016/j.carbon.2019.12.094.

(13) Paulus, G. L. C.; Wang, Q. H.; Strano, M. S. Covalent Electron Transfer Chemistry of Graphene with Diazonium Salts. *Acc. Chem. Res.* **2013**, *46* (1), 160–170. https://doi.org/10.1021/ar300119z.

(14) Ossonon, B. D.; Bélanger, D. Functionalization of Graphene Sheets by the Diazonium Chemistry during Electrochemical Exfoliation of Graphite. *Carbon N. Y.* **2017**, *111*, 83–93. https://doi.org/10.1016/j.carbon.2016.09.063.

(15) Greenwood, J.; Phan, T. H.; Fujita, Y.; Li, Z.; Ivasenko, O.; Vanderlinden, W.; Van Gorp, H.; Frederickx, W.; Lu, G.; Tahara, K.; Tobe, Y.; Uji-i, H.; Mertens, S. F. L.; De Feyter, S. Covalent Modification of Graphene and Graphite Using Diazonium Chemistry: Tunable Grafting and Nanomanipulation. *ACS Nano* **2015**, *9* (5), 5520–5535. https://doi.org/10.1021/acsnano.5b01580.
